# The Shrinking Fermi Liquid Scenario for Cuprates Under the Scrutiny of Optical Conductivity Measurements

**DOI:** 10.3390/ma17235849

**Published:** 2024-11-28

**Authors:** Sergio Caprara, Carlo Di Castro, Giovanni Mirarchi, Götz Seibold, Marco Grilli

**Affiliations:** 1 Istituto dei Sistemi Complessi—Consiglio Nazionale delle Ricerche and Dipartimento di Fisica, Università di Roma Sapienza, Piazzale Aldo Moro 5, 00185 Roma, Italy; sergio.caprara@roma1.infn.it (S.C.); carlo.dicastro@roma1.infn.it (C.D.C.); giovannimirarchi96@gmail.com (G.M.); 2 Institut für Theoretische Physik und Astrophysik, Universität Würzburg, Am Hubland, 97074 Würzburg, Germany; 3 Institut für Physik, Brandenburg Technical University Cottbus-Senftenberg, 03013 Cottbus, Germany; seibold@b-tu.de

**Keywords:** strange metal, cuprates, optical conductivity, shrinking Fermi liquid

## Abstract

In a recent paper [B. Michon et al., Nat. Commun. (2023) 14:3033], optical conductivity experiments in cuprate superconductors were shown to display scaling properties consistent with the Marginal Fermi Liquid theory. Here, we argue that the temperature regime studied in these experiments does not allow for distinguishing between Marginal Fermi Liquid and Shrinking Fermi Liquid. In the latter scenario, which we recently proposed and which applies near a quantum critical point, dynamical fluctuations of the order parameter with a short correlation length mediate a nearly isotropic scattering among the quasiparticles over the entire Fermi surface leading to strange metal behavior. If the damping of these nearly local fluctuations increases by decreasing the temperature, the Fermi liquid regime shrinks and the strange metal behavior is extended to the lowest temperatures. This Shrinking Fermi Liquid scenario has many similarities and some differences with respect to the Marginal Fermi Liquid theory. In particular, we show that the approximate scaling properties of the optical conductivity in some high-frequency regimes predicted by the Shrinking Fermi Liquid scenario account for a very good description of the experimental data.

## 1. Introduction

### 1.1. The General Framework: Strange Metallicity in Cuprates and Elsewhere

The anomalous properties of superconducting cuprates in their metallic state have always raised the issue of a new state of matter requiring a revision of the paradigmatic Landau Fermi liquid theory valid for ordinary metals. The occurrence of this ‘Strange Metal’ state (see, e.g., Refs. [1,2,3,4,5,6]), is a major theoretical and experimental challenge also because it occurs in many other systems like heavy fermions [7,8,9], pnictide superconductors [10,11], twisted bilayer graphene [12,13], and so on. At the moment, the main theoretical trends in this regard involve the coupling of the fermion quasiparticles with low-energy nearly local dynamical boson degrees of freedom. Recently, it has been proposed to represent these excitations by a so-called Sachdev–Ye–Kitaev model [14,15,16,17]. Similar results are obtained in Ref. [18], where the itinerant fermions are coupled to a two-level system. These boson excitations might arise from some small-momentum order parameter fluctuations (e.g., nematic) near a quantum critical point (QCP). Disorder is then responsible for turning these long-ranged excitations into local excitations.

On the other hand, the recent discovery [19,20] of nearly local charge density fluctuations (CDFs) in the form of ‘aborted’, very short-ranged, charge density waves (CDWs) have triggered the idea that CDFs are the sought-after nearly local degrees of freedom. For temperatures larger than the characteristic energy of CDFs $\omega_{0}^{CDF}$ a strange metal description is obtained [21]. Assuming that the damping of CDFs may increase when superconductivity is suppressed (usually by strong magnetic fields), $\omega_{0}^{CDF}$ decreases, thereby extending the strange metal behavior down to lower temperatures. This is the so-called Shrinking Fermi Liquid scenario [22,23,24]; see Section 1.2. In this framework, in order to understand the strange metal state, it is of primary relevance to extract from experiments indications on the low-energy dynamical excitations.

Concerning the dynamical aspects of the strange metal phase, the associated dynamical scattering rate can, in principle, be extracted from optical conductivity data. Motivated by possible quantum critical behavior, a number of experiments have been devoted to an analysis of the frequency-dependent scaling behavior [25,26,27,28], where early experiments [29,30] provided evidence for a power-law behavior $\sigma \left(\omega \right)∼1/{\left(i\omega \right)}^{\alpha }$, with a material and doping-dependent exponent α<1. More recently, it has been shown that such an apparent power-law behavior is in fact compatible with an underlying (local) self-energy which obeys ω/T scaling [28]. In particular, it has been claimed that a Marginal Fermi Liquid (MFL) structure [31] ImΣ(ω)∼βℏωcoth(βℏω), with $\beta \equiv {\left(kT\right)}^{-1}$, can account for the optical conductivity data in ${\mathrm{La}}_{2-p}$${\mathrm{Sr}}_{p}$${\mathrm{CuO}}_{4}$ (LSCO) at doping p=0.24, when both ℏω and kT are below a high-energy cutoff ∼0.2 eV. Moreover, the extracted seemingly logarithmic divergence of the optical mass upon reducing the temperature is then taken as an additional support for the analysis, since on the one hand this is compatible with the expected mass renormalization of fermions in a MFL state and on the other hand is consistent with the observed logarithmic enhancement of the specific heat in LSCO for the same doping value [32]. However, these conclusions are drawn from an analysis of optical conductivity data for temperatures T≳40 K so that the logarithmic divergence of the optical mass for T→0 is somewhat ambiguous. Furthermore, optical measurements are affected by a bunch of excitations living in the optical frequency range that can overshadow the contribution of the low-lying excitation that rules the DC transport.

In this paper, we will demonstrate that the Shrinking Fermi Liquid (SFL) theory [22,23,24] provides an alternative, and in some aspects more consistent, scenario in order to describe the optical conductivity data of cuprates. In particular, while MFL is a (very successful) phenomenological theory, SFL for cuprates has a definite microscopic character. This theory is based on the coupling of charge carriers to CDFs, which are observed and well-characterized boson excitations. The SFL is characterized by an energy scale $\omega_{FL}$, which separates Fermi Liquid (FL) (for $\omega <\omega_{FL}$) from non-FL (for $\omega >\omega_{FL}$) behavior. In our interpretation, the scattering is mediated by CDFs and the role of $\omega_{FL}$ is assumed by $\omega_{CDF}^{0}$. Since in some doping ranges, neither too far nor too close to the CDW quantum critical point (usually hidden in the superconducting region near optimal doping), $\omega_{FL}$ can become small at low temperatures [22,23], the scenario was termed SFL. Here, we will argue that the violation of the MFL paradigm in the optical conductivity below βℏω∼20 [28] is fully compatible with an underlying FL state with $\omega_{FL}\approx$ 5–10 meV comparable with $\omega_{0}^{CDF}$, the characteristic energy of CDFs [20]. In this regime, therefore, it is even not necessary to assume that this scale shrinks below the characteristic values observed for CDFs, as it is instead required to account for the linear-in-temperature resistivity and logarithmic specific heat, because these phenomena occur instead when superconductivity is suppressed down to a few kelvin. Nevertheless, this (not necessarily shrunk) FL state accounts for the temperature-dependent mass renormalization observed in optical experiments. Thus, the main purpose of this work is to show that optical experiments are not only compatible with MFL theory, but also SFL provides an acceptable (microscopic) alternative theory.

This paper is organized as follows: In Section 1.2, we recapitulate the main features of the SFL theory based on the exchange of CDFs. Subsequently, in Section 2, the evaluation of the optical conductivity within the SFL theory is undertaken. First, in Section 2.1, we discuss the energy spectra of CDFs as probed by RIXS, which, together with the phonon, paramagnon, and particle-hole contribution, constitute the dominant low-energy excitations in a large class of cuprate materials. Then, in Section 2.2, we report our calculations of optical conductivity within the SFL scenario, and in Section 3 we conclude our discussion.

### 1.2. The Shrinking Fermi Liquid Scenario

The last decade was characterized by impressive progress in Resonant Inelastic X-ray Scattering (RIXS), which unambiguously confirmed [33,34,35] that a strong tendency to form CDWs is present in underdoped and optimally doped cuprates, as predicted long ago [36,37,38,39,40]. More recently, RIXS experiments observed short-range dynamical CDFs, pervading most of the cuprates phase diagram [19,20]. These fluctuations have nearly the same periodicity as the CDWs, but, while they coexist in the underdoped region below the pseudogap crossover temperature $T^{*}$, CDFs alone are found up to the highest temperatures and dopings. In comparison to CDWs, the CDFs have a substantially shorter coherence length and a higher (but still rather small) characteristic energy $\omega_{0}^{CDF}∼$ 8–25 meV vs. $\omega^{CDW}∼$ 0–3 meV. Roughly speaking, owing to this distinct dynamical behavior, one can think of the CDFs as ‘aborted’ CDWs, which are nonetheless more robust both in temperature and doping. They have been detected over very broad parameter ranges and essentially in all classes of cuprates [41]. Remarkably, the abundant CDFs are the only excitations that are detected at relatively low energy in the optimal and overdoped regions at temperatures above $T_{c}$. Therefore, they are a natural candidate to explain the strange metal properties [1,14,15,42,43,44,45,46,47,48], most prominently the linear-in-temperature resistivity [49,50,51], in the metallic state of cuprates [21,22,23,24].

The main idea is that the CDFs fulfill two rather general sufficient conditions to obtain linear-in-temperature resistivity: (a) they are sufficiently short-ranged (i.e., nearly local in space) to provide a broad range of momenta in the scattering processes of quasiparticles. This first condition is needed in order to have the quasiparticles scattered all over the Fermi surface, thereby bypassing the objection of Ref. [52], which applies whenever the scattering is peaked at finite momenta, resulting in a partition of the Fermi surface in strongly scattered (hot) regions and weakly scattered (cold) regions; (b) the CDFs have sufficiently low energy to obey a semiclassical statistical distribution down to $T_{c}$. Owing to this latter condition, the Bose distribution of the collective scatterers is well-approximated by T/ω, resulting in a corresponding scaling behavior of the self-energy and, for ω=0, to a linear-in-temperature scattering rate, which is then responsible for the linear-in-temperature resistivity.

Our starting point is that the observed (and experimentally characterized) CDFs, near the critical wave vector $Q_{c}$, have a propagator of the typical form for fluctuations around Gaussian QCP [53], in the presence of Landau damping ∼γ (we set henceforth ℏ=1)

(1) $$D\left(q,\omega \right)=\frac{1}{M+\nu |q-Q_{c}{|}^{2}-\frac{\omega^{2}}{\bar{\Omega }}-i\gamma \omega }\;.$$

Here, *M* and ν represent the energy and stiffness of the CDFs, whereas $\bar{\Omega }$ is a crossover scale above which CDFs acquire a more propagating character.

Previous work [21] has shown that CDFs alone with γ∼1 can account for the anomalous MFL-like properties of cuprates well. In particular, these fluctuations have a quite short-ranged character and therefore have very weak momentum dependence, thus fulfilling the condition (b) mentioned above. At the same time, the characteristic energy can be as low as 8 meV [20] and can therefore also fulfill the condition (a) to give a linear-in-temperature inelastic quasiparticle scattering down to temperatures of order $T_{c}$. Also, the electron self-energy up to frequencies of order 0.15–0.2 eV was well-described by the coupling with CDFs.

Notice, instead, that the nearly momentum-independent interactions mediated by CDFs are unsuitable to mediate d-wave pairing. Moreover, they usually turn out to be mostly repulsive. This is contrary to the CDWs taking place closer to the CDW-QCP: CDWs do mediate a strongly attractive interaction and have a momentum structure that makes them favorable for d-wave superconductivity [54].

The success of the above theory in describing the strange metal above $T_{c}$ is limited by the fact that, by lowering the temperature, FL properties are recovered (i.e., the usual $T^{2}$ and $\omega^{2}$ behavior of the imaginary part of the fermion self-energy). This occurs below a temperature slightly smaller than the FL scale $\omega_{FL}=\omega_{CDF}^{0}=M/\gamma$, which is of order 40–60 K, at least. This is at odds with the observation of linear-in-temperature resistivity down to much lower temperatures, when superconductivity is suppressed by strong magnetic fields [55]. The condition (b) above prevents lowering $\omega_{FL}$ by decreasing $M∼\xi^{-2}$ approaching the QCP, because this would increase ξ (critical slowing down) and lead to strongly peaked momentum dependence of the fluctuation propagator. An alternative was recently proposed [22] to decrease the FL scale M/γ: assuming a temperature-dependent dissipation parameter γ∼log(1/T), the FL scale decreases *without* producing an increase in the spatial correlations (i.e., a reduction of *M*). The specific logarithmic form of γ(T) was phenomenologically inferred from the logarithmic increase in the low-temperature specific heat coefficient $C_{V}/T$ under a strong magnetic field [56]. In this case, the boson contribution to $C_{V}$ acquires a logarithmic contribution $C_{V}∼T\gamma \left(T\right)\log\left(1/M\right)$. While the standard QCPs may display a logarithmic temperature dependence from a vanishing mass of the fluctuations $M∼\xi^{-2}∼T$, here the system is assumed to be close, but not too much, to the QCP in order to have abundant fluctuations at finite (actually, rather small) correlation length and mass *M*. Then, the observed logarithmic behavior stems from the dissipation of the fluctuations γ(T) and is neither due to the standard critical behavior of the fluctuations nor to the diverging fermion quasiparticle mass $m^{*}$, as found instead in the MFL theory.

This scenario has been extended in two directions. In Ref. [23], a simple model was introduced, where the low-energy CDFs can not only decay in particle-hole pairs, but also in slow diffusive modes that always occur even in rather clean Drude metals whenever *T* is lower than the elastic scattering rate 1/τ. In two dimensions, this additional decay channel was shown to give rise to a logarithmic increase in the damping term γ. Of course, this is just one out of several other possibilities leading to an increasing damping, and further general effective mechanisms are still under investigation. On the other hand, a simplified fully local model of overdamped dispersionless Holstein phonons (ODHPs) was recently investigated [24] in order to gain an analytical understanding of the scattering rate and self-energy of a FL in the case of a temperature-dependent scale M/γ(T). In particular, within the SFL scenario, the electron self-energy turns out to be well-reproduced by the form

(2) ImΣR(ω,T)≃−λMγ2+ω2+(πkT)2−Mγ.

where $\lambda =g^{2}N\left(0\right)/M$ is a dimensionless coupling constant, with N(0) being the electron density of states at the Fermi level and *g* the coupling between the bosonic fluctuations and the fermionic quasiparticles. This should be compared with the self-energy customarily used in the MFL scenario

(3) ImΣRMFL(ω,T)≃−λω2+(πkT)2.

It is then clear that the frequency and temperature dependence of the quasiparticle self-energy turns out to be almost indistinguishable in the two scenarios whenever the FL scale M/γ is smaller than ω and/or *T*. In Ref. [24], it was also shown that this similarity persists at low temperatures if superconductivity is suppressed by very strong magnetic fields because γ=γ(T) increases when *T* is lowered, correspondingly reducing the FL scale. This obviously makes the experimental distinction between these two scenarios quite challenging (see the discussion in Section 3). Despite the strong similarities between our SFL scenario and the MFL scenario, some crucial differences are present. First of all, while it is well-known that the quasiparticle mass $m^{*}$ diverges logarithmically in *T* within the MFL scheme, in the SFL the quasiparticle mass is increased by the scattering with ODHPs, but it always stays finite. Another major difference is that in MFL, the dynamical quantities like electron self-energy and conductivity display an exact ω/T scaling, while such a property is broken by any finite M/γ in the SFL case. Of course, when M/γ becomes small, an approximate scaling may well be realized in the SFL case also. Thus, although the lack of scaling in SFL is not surprising, because the linear-in-temperature behavior of the scattering rate in SFL merely arises from the semiclassical statistical distribution function of the CDFs while the frequency dependence is related to their overdamped character, as a matter of fact, an approximate scaling still does take place for ω,T≫M/γ.

## 2. Optical Conductivity Within the SFL Scenario

The above summary of the SFL scenario indicates that CDFs alone can consistently account for the low-energy (thermodynamic and transport) properties of the metallic state of cuprates. The question naturally arises whether the SFL can also account for other spectroscopic properties of cuprates. In particular, it was recently shown [28] that optical experiments are in good agreement with MFL theory and, owing to the similarities between MFL and SFL, this latter may provide an equally good explanation for optical data.

### 2.1. The Experimental Characterization for Charge Density Fluctuations

**Figure 1:**
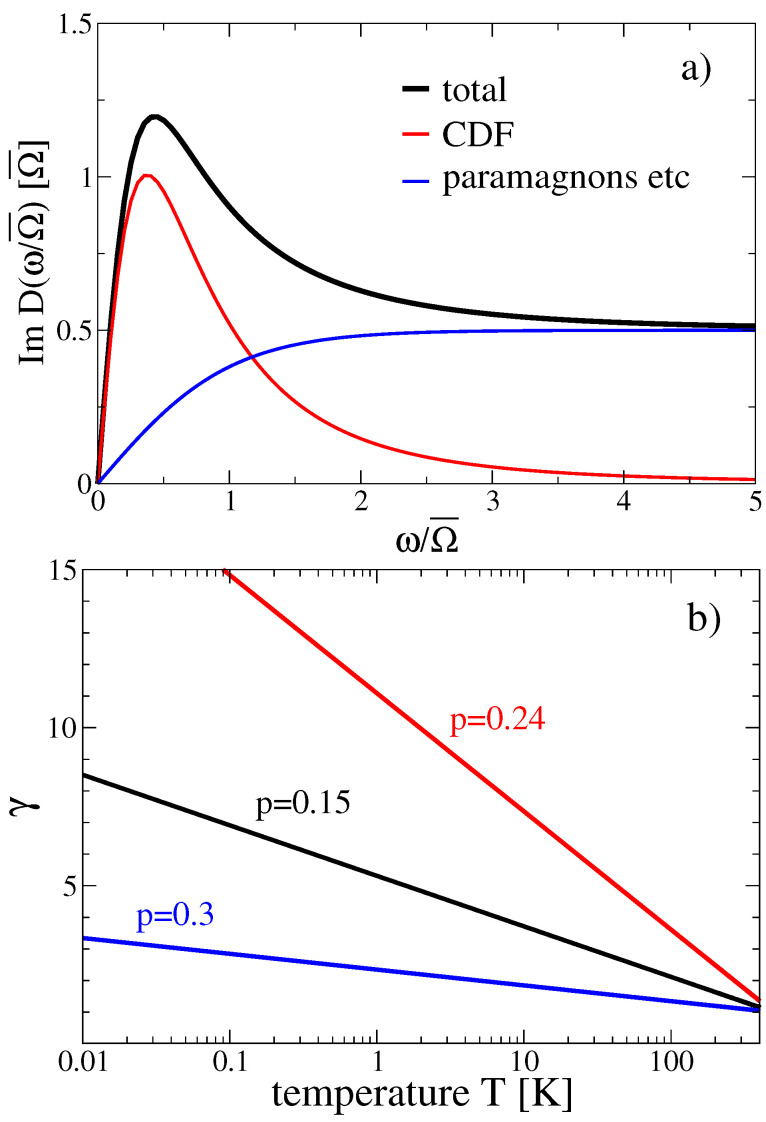
(**a**) The total spectral density (black, Equation (4)) is composed of the CDFs (red) and an effective higher energy contribution (blue) which encompasses phonon, particle-hole, and paramagnon excitations. Parameters α=0.5, $\omega_{p}/\bar{\Omega }=1$, $m/\bar{\Omega }=0.6$, γ=1.83 are evaluated from Equation (5) for T=300 K and doping p=0.24. (**b**) Temperature dependence of the γ parameter from Equation (5) for doping p=0.15 (black), p=0.24 (red), p=0.3 (blue), and further parameters are specified in the text.

The CDFs represented by Equation (1) have a spectral density, as shown by the red curve in Figure 1, and provide the microscopic basis for the theory at low energies. On the other hand, more standard excitations (phonon, particle-hole, and paramagnon excitations) are phenomenologically encoded as an additional flat contribution extending up to several fractions of eV. This is represented by the blue curve in Figure 1. Obviously, to describe the spectroscopic properties of cuprates at higher frequencies in optical experiments, one cannot neglect the whole set of excitations that are experimentally detected at higher energies (say from 0.05–0.5 eV). Thus, apart from the CDFs’ spectral density, we also consider the non-CDF excitations (namely phonon, particle-hole, and paramagnon excitations) as seen in the experimental RIXS spectral density of Figure 1c of Ref. [20]. Apart from their rather low characteristic energy scale $\omega_{0}^{CDF}$, CDFs are characterized by a structure factor that is broad in momentum and in frequency. The spectra, as a function of momenta, display a broad maximum centered at a $Q_{c}$, rather close to the CDW critical vector (indicating that these charge excitations mostly differ by their correlation length only). This peak is so broad (i.e., the associated spatial correlations are so short) that, as far as its effect on charge carriers is concerned, it can be well-approximated by a constant as for a fully local overdamped excitation and be represented by an ODHP [24]. A remarkably flat RIXS spectrum is also observed as a function of frequency for $q=Q_{c}$. The frequency-dependent RIXS spectra have then been fitted by several Gaussian contributions: slightly above the elastic peak (due to disorder in the surface, static deformation, and charge inhomogeneities) the CDFs give rise to a contribution from 8 to 30–50 meV. Excitations at higher energies are instead due to phonon, particle-hole, and paramagnon excitations, etc. Here, we employ a minimal model for such a multi-excitation RIXS spectrum by restricting to the CDFs and an effective higher energy contribution, cf. Figure 1, mimicking the experimental spectrum in Figure 1c of Ref. [20]. Notice also that, although the higher energy excitations have a composite nature, for the sake of brevity, we nickname as due to ‘paramagnon’ the whole flattish blue curve of Figure 1.

Therefore, we carried out the theoretical calculation of the cuprate optical properties by assuming the combined contribution of CDFs, schematized as an ODHP (red line in Figure 1), and of additional non-CDF excitations, represented by the blue line in Figure 1.

The total spectral density of these excitations reads

(4) D2(ω)≡ImD(ω)=γωM−ω2Ω¯2+γ2ω2+αΩ¯tanhωωp,

where the first term corresponds to the CDFs, Equation (1), with the modification that, due to the broadness of the peak, the momentum dependence is neglected and the resulting purely local CDFs are characterized by an effective mass *m*. For the temperature-dependent damping parameter γ, cf. Section 1.2, we adopt the phenomenological form of Ref. [23]

(5) $$\gamma =\gamma_{0}+A\left(p\right)\log\left[\max\left(\frac{1}{\tau T},1\right)\right],$$

with a doping-dependent prefactor A(p) specified in Equation (7) of Ref. [23]. Here, we focus mainly on the optical data from Ref. [28], which were obtained for LSCO at p=0.24. In this case, A(p=0.24)=0.087, 1/τ=500 K, and $\gamma_{0}=1$. Figure 1b shows the temperature dependence of γ for three selected doping values.

The second term in Equation (4) dominates the spectral density above a characteristic energy $\omega_{p}$, cf. Figure 1. Since $\omega_{p}$ is temperature independent, this contribution generally does not obey MFL scaling except in the high-frequency regime $\omega \gg \omega_{p}$.

**Figure 2:**
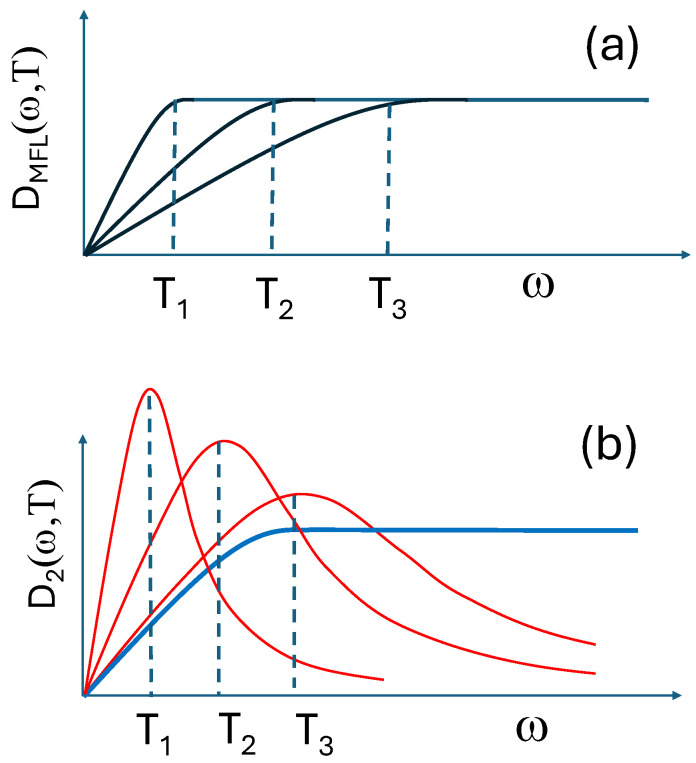
Sketch of the spectral density of the interaction mediators in (**a**) MFL and (**b**) SFL models. [In (**b**) the blue line is the ‘paramagnon’ contribution, while the red lines represent the CDF peak.

An important remark is in order now to make a comparison between SFL and MFL based on the specific forms of the interaction mediators. A MFL corresponds to a mediator spectral density of the form sketched in Figure 2a. In this case, the phenomenologically assumed broad spectral weight is either constant or linear in ω/T, thereby displaying a full scaling in this variable. On the other hand, the SFL interaction mediator, as already described above, is sketched in Figure 2b. In this latter case, none of the two contributions is scaling in ω/T: the flattish ‘paramagnon’ contribution because it does depend on frequency, but not on temperature, while the contribution of the CDF peak has a temperature-dependent slope at low frequency, but it also contains a scale M/γ(T), which only becomes small in specific conditions (low *T*, suppression of superconductivity, finite doping range) when γ(T) becomes large. Nevertheless, the sum of these two contributions conspires to mimic a MFL mediator spectral density, with a (nearly) constant flat part at large frequencies and a low energy part linear in ω with a slope that increases when M/γ decreases.

Then, it is not so surprising that SFL rather closely mimics the MFL, although no true ω/T scaling is formally present in it. This finding is also supported by previous theoretical calculations [57], where it was shown that a MFL optical conductivity might well be due to broad overdamped paramagnons of the form of Equation (4), showing that similar MFL optical behaviors can arise from somewhat different (but broad) excitation spectra.

### 2.2. Optical Conductivity Calculation

**Figure 3:**
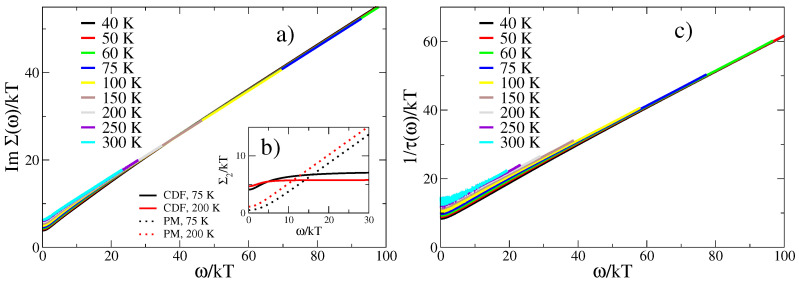
Scaling of the imaginary part of the self-energy (**a**) and of the optical scattering rate (**c**). Panel (**b**) shows the individual contributions to $\Sigma_{2}\left(\omega \right)$ arising from CDFs and the higher-energy, mainly paramagnetic (PM), fluctuations. Parameters: $g^{2}N\left(0\right)/\bar{\Omega }=1.0$, $\bar{\Omega }=30$ meV, α=1, $\omega_{p}/\bar{\Omega }=1$, $m/\bar{\Omega }=0.6$, κ=1 eV/(kΩ·cm).

In the case of local interactions, vertex corrections are perturbatively small in the calculation of optical conductivity. Therefore, it is customary to use the Allen approximation approach [58], where only the electron self-energy enters the calculation. Although vertex corrections might instead be present in the perturbative expansion of the self-energy, owing to the small-moderate value of the coupling between quasiparticles and CDFs [21] and phonons, we here assume that the one-loop approximation is sufficient. Then, we obtain the imaginary part of the one-loop electron self-energy as

(6) $$\Sigma_{2}\left(\omega \right)=-g^{2}N\left(0\right)\int d\omega^{\prime }\;D_{2}\left(\omega^{\prime }-\omega \right)\left[f\left(\omega^{\prime }\right)+b\left(\omega^{\prime }-\omega \right)\right],$$

with f(ω) and b(ω) denoting Fermi and Bose function, respectively, and we have assumed a constant electron density of states N(0). As can be seen from Figure 3a, $\Sigma_{2}\left(\omega \right)$ shows a remarkable scaling of $\Sigma_{2}\left(\omega /kT\right)$ above ω/kT∼20 although, as stated above, the individual contributions from CDFs and paramagnons do not scale. As illustrated in Figure 3b, this is due to a subtle cancellation between the CDFs and paramagnon self-energy shifts, which holds as long as both have comparable spectral density and the coupling of charge carriers $g^{2}N\left(0\right)$ to both excitations is the same. It should be noted that the approximate ratio of 2 between the intensities of low-energy CDFs and higher energy fluctuations in our model spectrum Figure 1 is also compatible with the RIXS spectrum in Figure 1c of Ref. [20]. Therefore, our choice of parameters which supports the cancellation between CDFs and paramagnon self-energy shifts is mirrored in the experiment.

Since the self-energy is momentum-independent, one can, after performing a Kramers–Kronig transformation for the real part $\Sigma_{1}\left(\omega \right)$, evaluate the optical conductivity within the Allen approach [58]

(7) $$\sigma \left(\omega +i\eta \right)=\frac{i\kappa }{\omega }\int d\omega^{\prime }\frac{f\left(\omega^{\prime }\right)-f\left(\omega^{\prime }+\omega \right)}{\omega -\Sigma \left(\omega^{\prime }+\omega +i\eta \right)+\Sigma^{*}\left(\omega^{\prime }+i\eta \right)}\;.$$

For a layered metal, like cuprates, $\kappa =e^{2}t/\left(\hbar d\right)$, where *t* is related to the kinetic energy and *d* is the interlayer spacing. If in Equation (7) ω is measured in eV, then $\kappa =O\left(1\right)\left[\mathrm{eV}/(k\Omega \cdot \mathrm{cm})\right]$.

For an isotropic FL, it has been shown [59] that Equation (7) can be represented in terms of a memory function M(ω) as [60,61,62,63]

(8) $$\sigma \left(\omega \right)=\frac{i\kappa }{\omega +M(\omega )}=\frac{\kappa }{\frac{1}{\tau (\omega )}-i\omega \frac{m^{*}\left(\omega \right)}{m_{0}}}\;.$$

From Equation (8), the optical relaxation time τ(ω) and optical mass enhancement $m^{*}\left(\omega \right)/m_{0}$ are then obtained as

1τ(ω)=κRe1σ(ω),m∗(ω)m0=−κIm1ωσ(ω),

when σ(ω) is evaluated from Equation (7). The optical relaxation time is shown in the lower panel of Figure 3 and displays a scaling behavior similar to that of the self-energy. In fact, in the limit ω/kT≫1, one can replace the Fermi functions in Equation (7) by their zero-temperature limit which yields the approximate result [28]

$$\sigma \left(\omega \right)=\frac{i\kappa }{\omega -2\Sigma (\omega /2)},$$

and therefore, $1/\tau \left(\omega \right)=2\Sigma_{2}\left(\omega /2\right)$. It is also interesting to observe that below ω/kT≈20, the scaling relation in Figure 3c is violated with decreasing temperature. This is exactly the behavior observed in the experimental data (Figure 5c of Ref. [28]) and corresponds to the crossover to a FL, below a temperature scale M/γ within our SFL scenario.

**Figure 4:**
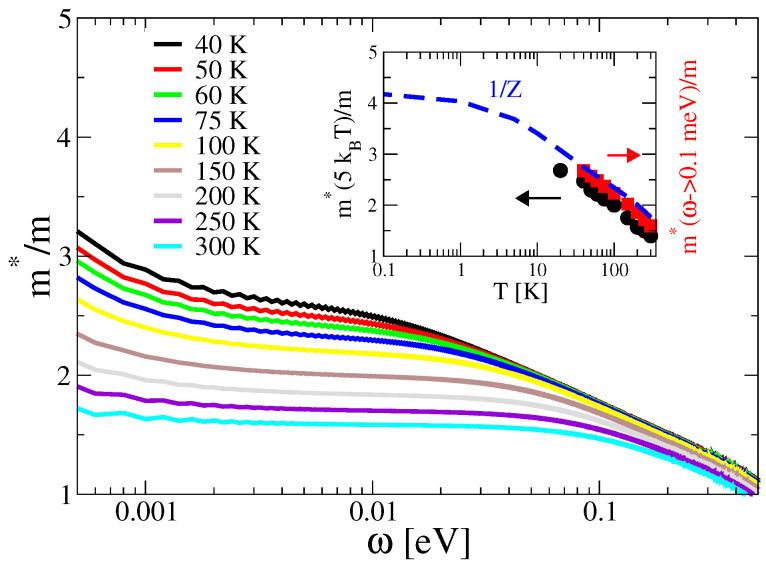
Main panel: optical mass m∗(ω)/m∼Re1/(ωσ(ω)), as extracted from the calculated optical conductivity. The inset shows the values as extracted at 5kT (black), extrapolated to zero frequency from the plateau at 10 meV (red), and the self-energy mass renormalization 1/Z (blue). Parameters: $g^{2}N\left(0\right)/\bar{\Omega }=0.6$, $\bar{\Omega }=30$ meV, α=1, $\omega_{p}/\bar{\Omega }=1$, $m/\bar{\Omega }=0.5$, γ=1.

Finally, Figure 4 shows the effective mass correction as a function of frequency for different temperatures. Following Ref. [28], we extract the mass correction at ω=5kT from the results and plot it as a function of temperature in the inset to Figure 4 (black circles). Moreover, we extract the mass correction at 10 meV and extrapolate it to ω=0.1 meV, which is shown by the red squares in the inset. Both higher-energy extrapolations of the mass correction show scaling behavior analogous to what is obtained in the experimental data of Ref. [28]. However, the low-energy mass renormalization

$$\frac{1}{Z}={\left.1-\frac{\partial \Sigma_{1}}{\partial \omega }\right|}_{\omega =0},$$

represented by the blue dashed line, converges to a constant in the limit T→0, in agreement with the fact that at the end of the day in this regime we are dealing with a FL [24].

### 2.3. Comparison with Experiments

We now proceed by comparing our calculations to the experiments of Ref. [28]. Before this, some remarks are in order. First of all, as mentioned in the introduction, the temperature range where optical conductivity experiments are carried out involves only minor corrections to the CDF damping parameter γ. Notice indeed that specific heat peaks [56], that we attribute to a bosonic contribution proportional to γ [22,23], only become visible below 10–15 K, and therefore the logarithmic corrections to it above 40 K are unessential (see also the discussion in the next section). This is why, to reduce the number of fitting parameters and keep the theoretical calculations as simple as possible, we disregard the *T*-dependent part of Equation (5). In this simplified framework, we also repeated the calculations that led to Figure 3 and Figure 4 and reported the results in the Appendix A. A second technical remark, which mostly concerns the calculations at low frequencies below, say, 20 meV, concerns the non-conserving character of the Allen scheme adopted in this work. This choice was adopted because of its simplicity and large use, that allows simpler comparisons with other theoretical calculations. However, it was shown long ago [53] that a non-conserving approach violating Ward identities (i.e., charge conservation laws), might overestimate the low-frequency optical absorption when purely electronic excitations (i.e., non-phononic) are considered. Here, we should keep in mind that Aslamazov–Larkin-like corrections and further vertex corrections (cf. diagram V in Figure 1c of Ref. [53]), might have some quantitative effect in the low-frequency regime for ω≃200 cm^−1^.

**Figure 5:**
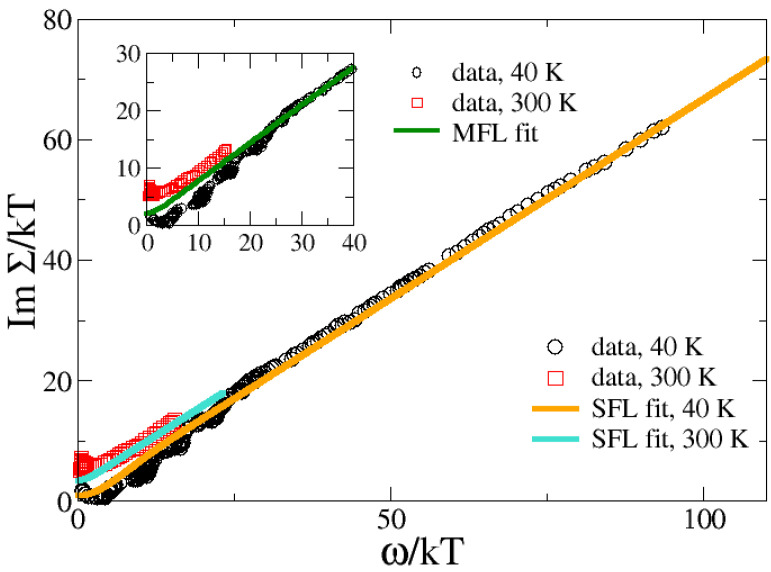
Comparison between theoretical calculations (see Section 2.2 with γ=2, solid lines) and optical conductivity data from Ref. [28] (circles and square symbols) at two different temperatures: T=40 K (orange curve and circles) and T=300 K (turquoise curve and squares). Parameters: $g^{2}N\left(0\right)/\bar{\Omega }=1.0$, $\bar{\Omega }=30$ meV, α=0.66, $\omega_{p}/\bar{\Omega }=0.5$, $m/\bar{\Omega }=0.6$. Inset: comparison between experimental data of Ref. [28] and the MFL theory (green curve), with ImΣMFL/kT=λ{(ω/kT)tanh(ω/kT)+π2[1+sech(ω/kT)]}, with the dimensionless coupling λ=0.66.

In Figure 5, we report the results of our calculation in the Allen approximation at low (T=40 K) and high (T=300 K) temperature for a constant value of $\gamma =\gamma_{0}=2$.

It is clearly visible that the fits are quite good even in the low-frequency regime, which, however, for the reasons mentioned above, is not quantitatively reliable due to the Allen approximation. Therefore, our SFL results have a quite good scaling character at ω/kT≳20, mimicking the true scaling of MFL, which is reported in the inset. Clearly, the experimental data violate MFL scaling for ω/kT≲20 which, however, is captured by the SFL scenario (within the Allen approximation). In this regime, the scattering is dominated by the CDF contribution, which results in a standard Fermi liquid at low temperatures or frequencies.

## 3. Discussion and Conclusions

Some general considerations are in order about the optical conductivity analysis carried out here. First of all, the definition of an optical conductivity requires that the electron system has some current dissipation mechanism, otherwise the total momentum conservation would force an identically vanishing conductivity. This is an important issue for those theories that involve the coupling to small momentum order parameter fluctuation (e.g., nematicity). In our case, instead, the SFL scenario involves large momentum scattering, so that umklapp processes are automatically included. Therefore, even disregarding the rich complications of umklapp processes [64], we can safely claim that our CDF-mediated scattering does allow for current dissipation, and quenched disorder can be naturally present, but it is not a crucial ingredient. We also notice that a further current dissipation mechanism is provided by the composite (electronic and phononic) nature of CDFs. It was shown long ago [53] that Ward identities, implementing current conservation in a Galilean-invariant system of electrons coupled to purely electronic CDWs, are instead violated as soon as the CDWs acquire a phonon component, thereby indicating that lattice dynamics can provide an additional current dissipation channel for the electronic subsystem.

**Figure A2:**
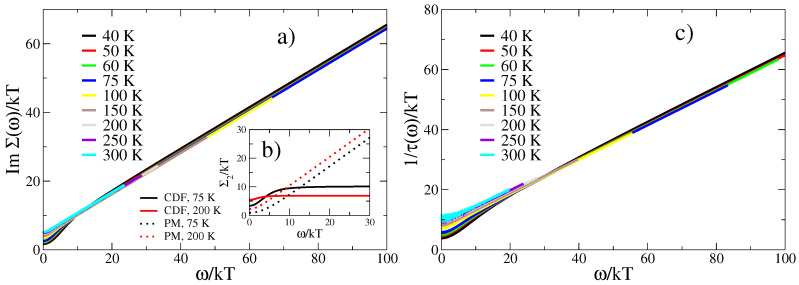
Scaling of the imaginary part of the self-energy (**a**) and of the optical scattering rate (**c**). Panel (**b**) shows the individual contributions to $\Sigma_{2}\left(\omega \right)$ arising from CDFs and the higher energy, mainly paramagnetic (PM), fluctuations. Parameters: $g^{2}N\left(0\right)/\bar{\Omega }=0.6$, $\bar{\Omega }=30$ meV, α=1, $\omega_{p}/\bar{\Omega }=1$, $m/\bar{\Omega }=0.5$, κ=1 eV/(kΩ·cm), γ=1.

The above analysis demonstrates that the experimentally observed RIXS spectrum [20], comprising low-energy CDFs plus higher-energy contributions from phonon, particle-hole, and paramagnon excitations contains the needed boson fluctuations to account for the optical properties of cuprates. Moreover, the (approximate) scaling properties identified in optical experiments are well reproduced by our model, although it ultimately describes a FL below an energy (temperature) scale M/γ. The enhancement of the specific heat coefficient in cuprates at very low temperature [32], T≲10 K forces our model [22] to invoke a shrinking of this energy scale through a growth of γ(T) from 1 to about 5–10 when *T* is lowered from 20 to 0.5 K. Notice that this substantial increase in γ is only relevant at low temperatures *and in the presence of very strong magnetic fields*, when the superconducting gap is suppressed, allowing for a sufficient growth of the electronic damping of the CDFs. This is the peculiar SFL effect extending the strange metal linear-in-temperature resistivity and the logarithmic specific heat at low *T*. On the other hand, the optical experiments discussed here [28] are carried out at higher temperatures $T>T_{c}∼40$ K, where the logarithmic increase in the damping plays a minor role and γ increases weakly (by a factor of two or so). Therefore, the FL energy scale M/γ stays non-negligible and leads to the observed violations of the scaling. Of course, elastic scattering from quenched impurities contributes as well, making it difficult to use the optical experiment to estimate the M/γ scale and discriminate it from elastic scattering. To do so, one should work in the unachievable regime (low *T* and very large magnetic fields), where γ grows substantially. In practice, for the present experiments, the increase of γ is not influential, as we show in Section 2.3 and in Appendix A, where we carry out the same analysis, also finding good agreement with the data when $\gamma =\gamma_{0}$. In fact, as shown in the Appendix A, an equivalent description of the optical data from Ref. [28] can be obtained *without* invoking Equation (5) and by fixing the damping parameter to a temperature-independent value γ=1 (which of course would be at odds with the explanation of specific heat data or linear-in-temperature resistivity down to low temperature). In any case, it is interesting to observe that the violation of the MFL paradigm is observed in the scaling of the optical scattering rate below ω/kT≈20 (cf. Figure 3, Figure 5 and Figure A2), which is therefore fully compatible with the crossover to a FL, as predicted by our theory. Of course, it may well be that the cuprates are in a genuine MFL state and that the mild scaling violations observed in experiments are due to elastic impurity scattering, the scaling being an intrinsic property. However, one should keep in mind that the scattering in an MFL approaches the unitary limit upon reducing temperature [65]. This should increase 1/τ(ω), whereas within our theory (and in the experiment), a reduction of 1/τ(ω) for small *T* (with respect to the linear relation) is found.

According to our analysis, one should be aware that the simpler explanation of a (shrinking) FL with low-energy (6–8 meV) CDFs plus phonons, particle-hole excitations, and paramagnons is enough to reproduce the data, without assuming any intrinsic scaling property. Of course the proximity to the charge-ordering QCP plays a role in producing abundant CDFs which display some quantum critical behavior at short length and time scales.

In conclusion, the present analysis shows that MFL and SFL are both internally consistent pictures calling for further theoretical and experimental investigations to discriminate between them. Comparison with other strange metals like CeCu${\mathrm{CeCu}}_{6-x}$(Au,Ag)_*x*_, pnictides, twisted bilayer graphene, and so on might also provide hints. In general, however, one has to face the fact that a clear-cut experimental distinction between the MFL and SFL scenarios is quite challenging. One first reason is that, as outlined in Figure 2 and related remarks, the composite spectral density (CDFs+phonons+paramagnons+…) closely resembles the one phenomenologically assumed by MFL theory. Therefore it is not surprising that the physical results are to a great extent similar. Moreover, the proximity to a QCP provides abundant CDFs at rather low energy for the SFL scenario which, therefore, for *T* larger than $\omega_{FL}$, becomes similar to MFL theory. The same holds for the high-frequency dependence $\omega >\omega_{FL}$, where the nearly constant composite spectral density is MFL-like as a matter of fact. Moreover, experimental probes involving the integration of degrees of freedom generically wash out small differences in the spectral densities both of fermions and bosons (see, e.g., Ref. [57]). Thus, given the observed CDF spectral density, the MFL and the SFL scenario are hardly distinguishable from photoemission or other spectroscopic tools. Even shot-noise analyses, like that recently carried out in heavy fermions [66], may prove to be not fully conclusive. If such an analysis is carried out in cuprates, at low temperatures and strong magnetic fields, we expect a reduction in shot noise due to the quasiparticle scattering with the CDFs. For some specific doping ranges, the CDFs should display a very low $\omega_{FL}$ producing shot-noise reduction in a way similar to what phonons do at higher temperatures. Also, in this case, however, the shot-noise reduction might as well be interpreted within the MFL scenario.

The real benchmark for the SFL scenario would be to test directly the behavior of the low-frequency spectral density of CDFs at low *T in the absence of superconductivity* checking whether or not the M/γ scale decreases because of an increased damping γ(T). This would require, e.g., RIXS experiments under strong magnetic fields of several tens of teslas to suppress superconductivity (remember that in the presence of superconductivity the parameter γ is obviously small since the Landau damping is suppressed by the superconducting gap). Such experiments are presently out of reach for obvious technical reasons. Reducing $T_{c}$ by, e.g., Zn impurities is another option, but one should keep in mind that these might perturb the CDFs by providing pinning centers.

As far as the violations to the scaling of low-frequency optical spectra is concerned, we wish to add here a final remark. One should be warned that scaling properties of optical spectra should be discussed with caution [53], because many processes can affect the low-frequency optical response of bad metals, partially masking or modifying the contributions arising from self-energy corrections dressing the charge carriers, that were discussed in this piece of work. The possibility exists that collective excitations may directly contribute to the optical response [67], their coupling to the electromagnetic field being possibly mediated by charge carriers [68]. The contribution to the optical response of processes with CDFs alone as intermediate (virtual) states are currently under investigation. Increasing theoretical evidence indicates that slow/overdamped collective excitations can introduce displaced (finite-frequency) peaks in the optical response of bad metals [67,69,70,71]. Unless an experiment can access the very low-frequency regime, the shoulders of these displaced peaks can be easily mistaken for anomalously large Drude peaks [72,73,74].

## Figures and Tables

**Figure A1:**
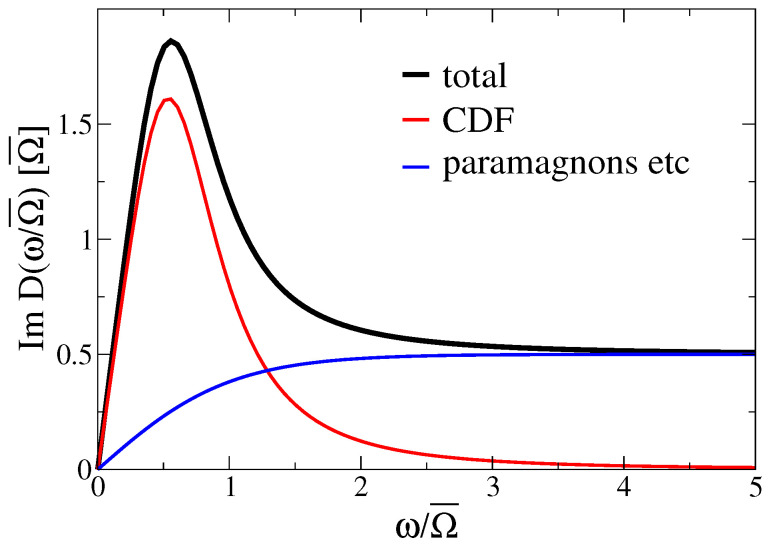
The total spectral density (black, Equation (4)) is composed of the CDFs (red) and an effective higher energy contribution (blue), which encompasses phonon, particle-hole, and paramagnon excitations. Parameters: α=1, $\omega_{p}/\bar{\Omega }=1$, $m/\bar{\Omega }=0.5$, γ=1 (see text).

**Figure A3:**
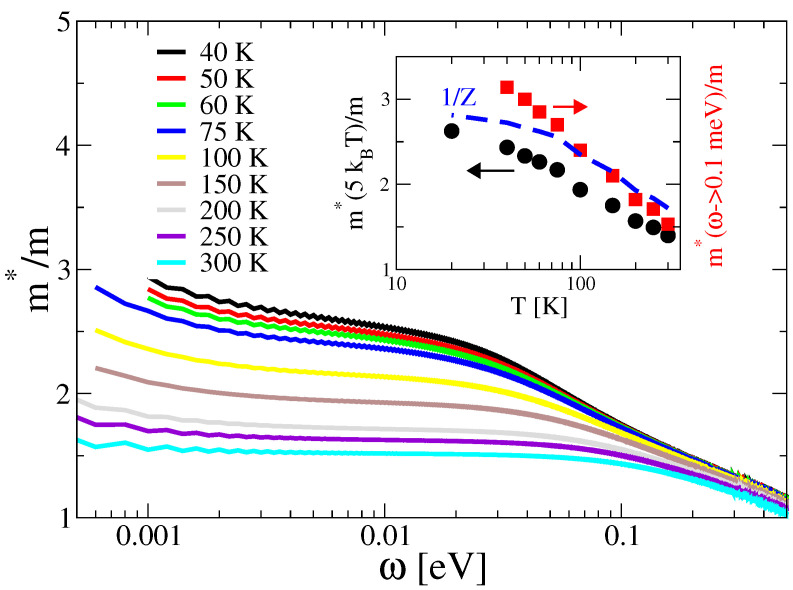
Main panel: Optical mass m∗(ω)/m∼Re1/(ωσ(ω)), as extracted from the calculated optical conductivity. The inset shows the values as extracted at 5 kT (black), extrapolated to zero frequency from the plateau at 10 meV (red), and the self-energy mass renormalization 1/Z (blue). Parameters: $g^{2}N\left(0\right)/\bar{\Omega }=0.6$, $\bar{\Omega }=30$ meV, α=1, $\omega_{p}/\bar{\Omega }=1$, $m/\bar{\Omega }=0.5$, γ=1.

## Data Availability

The original contributions presented in the study are included in the article, further inquiries can be directed to the corresponding author.

## Appendix A. Results for Constant *γ* = 1

As discussed in Section 3, the optical data from Ref. [28] are obtained in a frequency and temperature regime where the logarithmic corrections to the damping factor are negligible. Therefore, we report in this Appendix A the results of a fixed γ=1, starting again from a propagator, which is shown in Figure A1 and, in contrast to Figure 1, does not depend on temperature.

In this case, the temperature dependence is solely given by the Fermi and Bose functions, which enter the evaluation of the self-energy Equation (6) and the optical conductivity Equation (7). The resulting scaling behavior of ImΣ(ω) and 1/τ(ω) is shown in Figure A2 and can be compared to the result of Figure 3 obtained within the SFL scenario. Small differences can be observed at low energies, related to the deviation from FL behavior. However, these are far beyond the present experimental accuracy.

Finally, Figure A2 reports the mass renormalization for the scenario with fixed γ=1 which again is close to Figure 4, in particular one observes the crossover to a FL at low temperatures in both cases.
